# Light-driven diel oscillations in microbial metabolism underpin estuarine biogeochemical resilience

**DOI:** 10.1093/ismeco/ycaf216

**Published:** 2025-11-21

**Authors:** Zhuoli Zhao, Jialing Li, Ziqi Peng, Xiaoqing Luo, Li Duan, Zhiliang Lin, Pandeng Wang, Wenjun Li, Hongchen Jiang

**Affiliations:** School of Ocean Sciences, China University of Geosciences (Beijing), Beijing 100083, China; State Key Laboratory of Biocontrol, School of Life Sciences, Sun Yat-Sen University, Guangzhou 510275, Guangdong, China; State Key Laboratory of Biocontrol, School of Life Sciences, Sun Yat-Sen University, Guangzhou 510275, Guangdong, China; State Key Laboratory of Biocontrol, School of Life Sciences, Sun Yat-Sen University, Guangzhou 510275, Guangdong, China; State Key Laboratory of Biocontrol, School of Life Sciences, Sun Yat-Sen University, Guangzhou 510275, Guangdong, China; State Key Laboratory of Biocontrol, School of Life Sciences, Sun Yat-Sen University, Guangzhou 510275, Guangdong, China; State Key Laboratory of Biocontrol, School of Life Sciences, Sun Yat-Sen University, Guangzhou 510275, Guangdong, China; State Key Laboratory of Biocontrol, School of Life Sciences, Sun Yat-Sen University, Guangzhou 510275, Guangdong, China; School of Pharmacy, Shenzhen University Medical School, Shenzhen University, Shenzhen 518055, Guangdong, China; State Key Laboratory of Biocontrol, School of Life Sciences, Sun Yat-Sen University, Guangzhou 510275, Guangdong, China; School of Ocean Sciences, China University of Geosciences (Beijing), Beijing 100083, China; School of Life Sciences, Henan University, Kaifeng 475004, China

**Keywords:** diel light cycle, planktonic prokaryotes, metabolic networks, biogeochemical cycling, Pearl River estuary

## Abstract

Diel light cycles profoundly influence estuarine biogeochemical processes, yet the mechanistic responses of planktonic prokaryotic communities to these rhythmic cues remain incompletely understood. This study employed an integrative multi-omics approach—combining high-frequency sampling, 16S rRNA gene sequencing, metagenomics, and metatranscriptomics—to elucidate diel dynamics in microbial diversity, interaction networks, and metabolic functions in the Pearl River Estuary. The results revealed significant temporal partitioning in microbial organization: nocturnal communities exhibited higher α-diversity and formed more densely connected co-occurrence networks, indicative of enhanced heterotrophic processes, whereas daytime assemblages were dominated by *Cyanobacteria* (particularly *Synechococcales*) with enriched pathways for photoautotrophic carbon fixation and nitrogen assimilation. Metabolic profiling further demonstrated distinct diel oscillations in key biogeochemical processes, including daytime enhancement of Calvin cycle-mediated CO_2_ fixation and nocturnal upregulation of dissimilatory sulfate reduction. Network topology analysis showed that nighttime communities displayed increased clustering coefficients and reduced path lengths, suggesting more efficient resource utilization under dark conditions. Through reconstruction of 786 metagenome-assembled genomes, we identified *Cyanobiaceae* as key mediators of diel carbon and nitrogen transformations, while diverse heterotrophic taxa facilitated nighttime nutrient remineralization. This study provides mechanistic insights into how light-driven diel oscillations shape microbial metabolic partitioning and ecological interactions, advancing our understanding of the temporal dynamics that underpin biogeochemical resilience in estuarine ecosystems.

## Introduction

Estuaries, as highly dynamic interfaces bridging terrestrial and marine realms, are acknowledged as pivotal global hotspots of biogeochemical cycling owing to their elevated nutrient fluxes, pronounced hydrodynamic gradients, and remarkably diverse microbial consortia [1, 2]. Characterized by steep spatial and temporal environmental heterogeneity—including variations in salinity, temperature, and irradiance—these ecosystems impose stringent selective pressures on microbial assemblages, shaping their composition and function [3, 4]. Among the myriad environmental drivers, diel light cycles—dictated by Earth’s rotation—exert profound influence over microbial physiology, metabolic pathways, and community dynamics, especially within the photic zone where light functions dually as an energy source and a regulatory signal [5, 6]. Despite the recognized importance of these diel oscillations in molding estuarine biogeochemistry, the underlying mechanistic responses of planktonic prokaryotic communities to such rhythmic environmental variations remain insufficiently elucidated [7, 8].

Emerging research illuminates significant diel oscillations in microbial community structure, gene expression profiles, and metabolic activity across both marine and lacustrine ecosystems [9, 10]. For example, conspicuous diel successions involving *Cyanobacteria* and heterotrophic bacteria have been observed in open ocean settings, with photoautotrophs exhibiting dominance during illuminated periods and heterotrophs facilitating organic matter remineralization during darkness [11, 12]. Analogously, riverine systems demonstrate diel fluctuations in microbial activity correlated with light-mediated primary production and nutrient assimilation [13, 14]. However, estuarine environments pose distinct challenges stemming from their pronounced physicochemical gradients, tidal mixing dynamics, and susceptibility to anthropogenic influences, factors capable of either accentuating or mitigating diel microbial responses relative to more stable aquatic settings [15, 16]. Therefore, simplistic extrapolation of findings derived from open ocean or freshwater systems onto estuaries carries the risk of oversimplifying the intricate microbial adaptations to rhythmic environmental forcing.

The Pearl River Estuary (PRE), a prominent subtropical estuarine system in China, embodies these complexities. Marked by elevated nutrient loading, intense tidal currents, and sharp ecological gradients, the PRE harbors a highly dynamic microbial community that plays a crucial role in mediating essential biogeochemical processes, including carbon fixation, nitrogen cycling, and sulfur transformation [17, 18]. While prior investigations have documented shifts in the taxonomic composition of PRE microbial communities along spatial and seasonal gradients [19, 20], the temporal partitioning of microbial functions influenced by diel light cycles remains largely unexamined. Specifically, the mechanisms by which rhythmic light availability shapes the metabolic specialization, network interactions, and overall ecological strategies of planktonic prokaryotes within this system remain obscure. Addressing this knowledge deficit is paramount for comprehending the resilience of estuarine ecosystems in the face of environmental disturbances and for forecasting how microbial processes will respond to global-scale alterations such as eutrophication and climate warming.

Drawing upon the burgeoning body of evidence indicating that diel light cycles orchestrate the temporal partitioning of microbial activities in marine systems [21, 22], we propose the hypothesis that planktonic prokaryotic communities within the PRE exhibit distinct diel dynamics across three fundamental parameters: (i) community composition, characterized by shifts in taxonomic structure driven by light-dependent resource competition; (ii) interaction networks, featuring diurnally fluctuating connectivity patterns that mirror metabolic interdependencies; and (iii) metabolic functions, manifested as the partitioning of anabolic processes (e.g., carbon fixation) and catabolic processes (e.g., organic matter degradation) between daytime and nighttime periods. To evaluate this hypothesis, we implemented a 48-hour high-resolution sampling program within the PRE, employing an integrated approach combining 16S rRNA gene amplicon sequencing, metagenomics, and metatranscriptomics to decipher diel variations in microbial diversity, co-occurrence networks, and metabolic capabilities.

Our study addresses a significant gap in microbial ecology by unraveling how diel light cycles influence the functional partitioning and ecological strategies of prokaryotic communities in estuarine environments. Through the application of integrated multi-omics approaches, we aim to (i) quantify diel variations in α-diversity and community composition, (ii) reconstruct microbial co-occurrence and interaction networks to identify keystone taxa and functional modules modulated by light cycles, and (iii) analyze diel shifts in transcriptional activity across key biogeochemical pathways involved in carbon, nitrogen, and sulfur cycling.

## Materials and methods

### Study site and sample collection

The PRE, located in southern China, represents a dynamic subtropical estuarine system characterized by complex hydrodynamics, pronounced nutrient fluxes, and active biogeochemical cycling. Sampling was conducted in the PRE at station T1 (22.09°N, 113.76°E), located in the mid-estuarine mixing zone where freshwater from the Pearl River meets saline water from the South China Sea under mesohaline–polyhaline conditions [23]. This area represents a transitional mixing zone that is widely recognized as biogeochemically active and hydrodynamically well mixed over short spatial scales. Although estuarine systems are inherently heterogeneous, this mid-estuarine zone is characterized by relatively weak vertical stratification and moderate nutrient concentrations, making it suitable for capturing diel microbial dynamics under comparatively stable environmental conditions. Sampling was performed over a 48-hour interval commencing at 15:00 local time (GMT + 8) on July 15, 2020. Using a conductivity-temperature-depth (CTD) rosette sampler equipped with 10 L Niskin bottles, surface seawater (~1 m depth) was collected every 4-hour intervals from a fixed location, yielding a total of 12 temporal points, resulting in the acquisition of 24 discrete samples (particle-attached (PA) and free-living (FL) fractions at each point). At each time point, ~10 L of surface water were collected. Concurrently, the CTD profiler recorded *in situ* environmental parameters, including temperature, salinity, dissolved oxygen, turbidity, depth, and pH (Table S1). Estuary water was subjected to immediate filtration subsequent to collection, employing a sequential size-fractionation methodology. Initially, the water was passed through a 3.0 μm Millipore polycarbonate filter to collect PA microbial communities. The resultant filtrate was then passed through a 0.2 μm Pall Life Sciences polyethersulfone membrane to collect FL microbial populations. Following filtration, filters were immediately flash-frozen in liquid nitrogen and subsequently stored at −80°C pending laboratory analyses. Additionally, a supplementary aliquot of ~100 ml of seawater from each sample was stored at 4°C in the dark. A subsample was treated with a 4% paraformaldehyde solution for enumeration of microbial cells via flow cytometry. The remaining volume was transported to the laboratory for comprehensive chemical analyses, including dissolved organic carbon (DOC), total nitrogen (TN), nitrite (NO₂^−^), nitrate (NO₃^−^), and ammonium (NH₄^+^).

### RNA and DNA extraction and sequencing

Total RNA was extracted from 24 membrane filters using the PowerSoil® Total RNA Isolation Kit (MO BIO) in accordance with the manufacturer’s standard protocol. Following RNA extraction, the same samples were subjected to DNA extraction using the PowerSoil® DNA Elution Kit (MO BIO). The concentration and purity of genomic DNA and total RNA were assessed with a NanoDrop spectrophotometer (Thermo Fisher Scientific). Qualified samples were then forwarded to Majorbio Bio-Pharm Technology Co., Ltd. (Guangzhou, China) for amplicon, metagenomic, and metatranscriptomic sequencing. Reverse transcription of RNA samples was performed to generate cDNA, which was subsequently used as templates for amplification of the V4-V5 hypervariable regions of the prokaryotic 16S rRNA gene using primers 515F-Y (5’-GTGYCAGCMGCCGCGGTAA) and 926R (5’-CCGYCAATTYMTTTRAGTTT) [24]. Amplicon sequencing was performed on the Illumina NovaSeq 6000 PE250 platform, while metagenomic and metatranscriptomic libraries were sequenced on the Illumina HiSeq PE150 platform.

### Microbial community analysis

Amplicon sequence data were processed using the USEARCH pipeline. Paired-end reads were merged, and primer sequences were trimmed using CUTADAPT v2.4 [25]. Sequences with a maximum expected error exceeding 1.0 or a length below 350 bp were discarded. The remaining high-quality sequences were denoised using the Unoise3 algorithm with default parameters, to generate zero-radius operational taxonomic units (zOTUs). Taxonomic classification of zOTUs was performed using a Naïve Bayes classifier [26] trained against the SILVA 132 database within the QIIME2 platform (qiime2–2022.11) [27].

A zOTU table was constructed by mapping trimmed sequences to representative sequences of the zOTUs. To account for variation in sequencing depth, the dataset was rarefied using the rrarefy function in the vegan R package, normalizing all samples to the minimum observed sequence count (13 655 reads). Rarefaction was performed jointly across all particle-associated (PA) and FL samples to ensure comparability of diversity metrics between groups. This resulting dataset comprised 327 720 high-quality sequences, which were utilized for subsequent analyses.

Alpha diversity was quantified using the Hill numbers implemented in the renyi function of the vegan package [28]. Indices included species richness (q = 0), the exponential of Shannon entropy (q = 1), and the inverse Simpson index (q = 2), which increasingly weights dominant species [29]. Community similarity between samples was assessed using Bray–Curtis dissimilarity, and multidimensional scaling (MDS) analysis was performed using the metaMDS function in vegan.

### Co-occurrence network analysis

Taxonomic annotation was performed on the zOTU table to determine bacterial community composition at different taxonomic levels. To ensure network robustness and minimize spurious correlations, zOTUs present in less than one-third of the samples were excluded from network construction. Microbial interaction networks were constructed based on a correlation matrix generated by SparCC [30]. Node roles within the network were characterized by calculating within-module connectivity (Zi) and among-module connectivity (Pi), following the approach of Guimerà and Amaral [31]. Specifically, nodes were classified into four distinct types: module hubs (Zi > 2.5, Pi i0.62), connectors (Zi ≤ 2.5, Pi >0.62), network hubs (Zi > 2.5, Pi >0.62), and peripheral nodes (Zi ≤ 2.5, Pi <0.62) [32]. Nodes with elevated Zi or Pi values, excluding those classified as peripheral, were considered potential keystone taxa due to their likely significant ecological roles.

Network properties, including key topological characteristics, were calculated using the igraph R package [33]. Network visualization and graphical representation were performed using Gephi (version 0.10.1) [34].

### Identification and quantification of carbon, nitrogen, and sulfur metabolism genes

Low-quality sequences and adapter contamination in the metagenomic data were removed using fastp (v0.20.0) with the following parameters: -n 0 -l 30 —cut_front —cut_right —cut_window_size 4 —cut_mean_quality 30 [35]. Clean reads were assembled using MEGAHIT v1.2.9 with parameters —k-min 27 —k-max 127 —k-step 20 —min-contig-len 500 [36]. Open reading frames (ORFs) were predicted from assembled contigs in meta mode using Prodigal v2.6.3 (−m -p meta) [37].

The predicted ORFs were clustered using CD-HIT-EST v4.8.1 at 95% sequence identity (−c 0.95 -n 10 -G 0 -aS 0.9 -G 1 -d 0 -T 20 -M 0) to generate a non-redundant gene catalog [38, 39]. Functional annotation of the ORFs was conducted using DIAMOND v0.9 against the KEGG and Carbohydrate-Active enZYmes (CAZy) databases with parameters -k 1 -e 0.00001 -p 15 -query-cover 30 [40].

Metatranscriptomic reads were also quality-filtered using fastp (v0.20.0) with the parameters -n 0 -l 30 —cut_front —cut_right —cut_window_size 4 —cut_mean_quality 30 [35]. Ribosomal RNA sequences were removed using RiboDetector. The high-quality metatranscriptomic reads were subsequently mapped to the non-redundant ORF catalog using BBMap with a minimum identity threshold of 95% (minid = 0.95) [41]. Transcript abundance was calculated and expressed as Reads Per Kilobase of exon model per Million mapped reads (RPKM).

To assess diel patterns in functional gene expression, samples were systematically classified into “day” and “night” categories. For each sample, the transcript abundances of functionally annotated ORFs were summed within their respective metabolic categories. Genes annotated through either KEGG Orthology or CAZy annotations were aggregated if they assigned to the same functional category, with their RPKM (Reads Per Kilobase of transcript per Million mapped reads) values were combined to calculate total pathway-level expression profiles.

### Genome binning, dereplication, and annotation

Metagenome-assembled genomes (MAGs) were recovered from each sample following previously described methods [18, 19]. Briefly, MetaBAT2 was employed with default parameters to bin scaffolds based on differential coverage across samples and tetranucleotide frequency, thereby reconstructing MAGs [42]. MAG quality was assessed using CheckM v1.0.12 [43], and potential contaminants were identified and removed using RefineM v0.0.25 [44].

High- to medium-quality MAGs (completeness >70%, contamination <10%) from all samples were pooled and dereplicated using dRep v2.6.2 [45]. Taxonomic classification was performed using GTDB-Tk v1.2.0 [46]. ORFs within MAGs were predicted using Prodigal v2.6.3 with default settings [37], and annotated by searching against reference databases using DIAMOND v0.9, as described previously [40].

The quantification of transcript abundance for carbon, nitrogen, and sulfur metabolism genes within each MAG was performed as described in Section *Identification and quantification of carbon, nitrogen, and sulfur metabolism genes*.

### Statistical analysis

Wilcoxon rank-sum tests were employed to interrogate disparities in measured environmental parameters and α-diversity between diurnal and nocturnal cohorts. Permutational multivariate analysis of variance (PERMANOVA), predicated on Bray–Curtis dissimilarities, was performed to elucidate the impact of diel photoperiods on microbial community architecture. Differential gene expression analysis was conducted utilizing the DESeq2 package [47], with genes deemed significantly differentially expressed if they exhibited an absolute log_2_ fold change two coupled with an adjusted *p*-value ≤0.05.

## Results

### Environmental stability during diel sampling

To ensure that observed diel variations in microbial community structure and metabolic activities were not confounded by physicochemical fluctuations, we compared the measured environmental parameters between daytime and nighttime samples (Table S1). Parameters included temperature, salinity, turbidity, pH, dissolved oxygen (DO), DOC, TN, NO₂^−^, NO₃^−^, NH₄^+^, total phosphorus (TP) and tidal height. Tidal height information was obtained from the National Marine Data Center (https://mds.nmdis.org.cn/).

Wilcoxon rank-sum tests showed no statistically significant differences (*P* > 0.05) in the measured environmental parameters (e.g. temperature, salinity, pH, DO, turbidity) between the day and night sample groups (Table S2). These results indicate that the physicochemical environment remained relatively stable during the 48-hour sampling period. Therefore, the diel changes in microbial diversity and transcriptional profiles identified in this study primarily reflect biological responses to periodic light–dark cycles, rather than short-term hydrological or nutrient variations.

### Microbial community diversity and structure under diel light cycles

**Figure 1:**
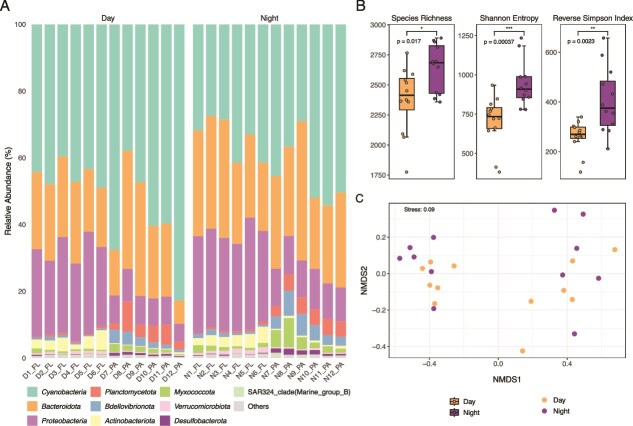
Composition and diversity of planktonic prokaryotic microbial communities under diel light cycles in the Pearl River estuary. (A) Relative abundances of the top 10 most dominant bacterial phyla, with less abundant phyla grouped and labeled as “others.” (B) alpha diversity indices—Including species richness, Shannon entropy, and inverse Simpson index—Of microbial communities sampled during day and night. Asterisks denote levels of statistical significance (*P* < 0.05, ^**^P < 0.01, ^***^P < 0.001). (C) Non-metric multidimensional scaling (NMDS) plot based on Bray-Curtis dissimilarities illustrating community compositional differences across diel groups.

Amplicon sequencing targeting the 16S rRNA gene, performed on 24 samples, resolved a total of 35 phyla, 60 classes, 149 orders, 220 families, and 326 genera. The relative abundances of *Cyanobacteria*, *Bacteroidota*, and *Proteobacteria* varied substantially, ranging from 5.2% to 82.8% across all samples. These phyla exhibited mean relative abundances of 45.8%, 25.2%, and 18.5%, respectively (Fig. 1A). Notably, *Cyanobacteria* were more abundant during daylight hours (52.3%) compared to nocturnal periods (39.3%), whereas *Bacteroidota* (22.1% during the day vs. 28.6% at night) and *Proteobacteria* (16.9% during the day vs. 20.0% at night) showed elevated abundance at night.

Analysis of alpha diversity indices showed statistically significant diel variations (*P* < 0.05, Fig. 1B). Species richness, Shannon entropy, and the inverse Simpson index were all significantly greater in samples collected at night, indicating an increased taxonomic complexity and diversity within the microbial community during this period. However, non-metric multidimensional scaling (NMDS) ordination based on Bray–Curtis dissimilarity matrices did not reveal a significant difference in overall microbial community composition between diurnal and nocturnal groups (Adonis, R^2^ = 0.0853, *P* > 0.05, Fig. 1C).

### Microbial co-occurrence networks under diel light cycles

**Figure 2:**
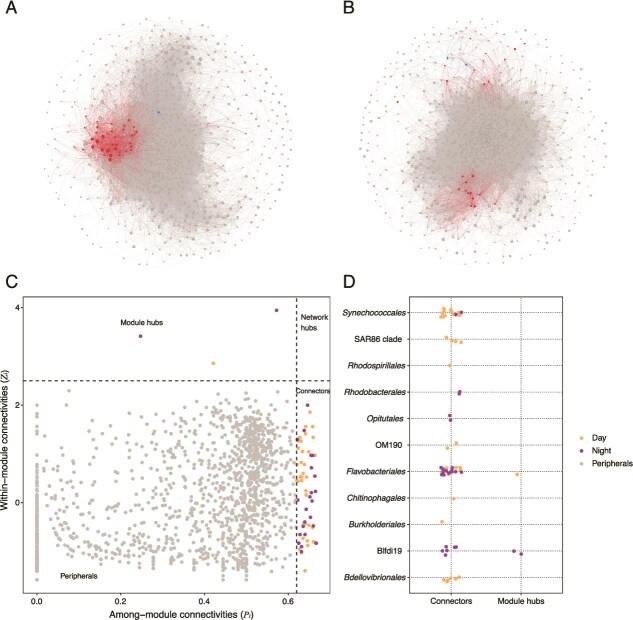
Molecular ecological networks reveal diel shifts in planktonic prokaryotic community structure. (A) Co-occurrence network of the planktonic prokaryotic community during the photic period. (B) Co-occurrence network during the aphotic period. In both networks, node size is scaled to the log₂-transformed relative abundance of each zero-radius operational taxonomic unit (zOTU). Node color designates ecological role: Peripheral nodes are shaded gray, connector nodes red, and module hub nodes blue. (C) Zi–pi plot illustrating the distribution of ecological roles, with orange and purple representing diel and nocturnal networks, respectively. (D) Taxonomic composition (order level) of connector and module hub nodes identified in each network.

Microbial interaction networks constructed using SparCC revealed significant differences in community structure and connectivity between day and night in the Pearl River Estuary (Fig. 2A, B). The night network (Fig. 2B), with 900 nodes and 57 119 edges, exhibited greater complexity and connectivity than the day network (Fig. 2A, 735 nodes, 33 474 edges), suggesting more extensive microbial interactions during nighttime (Table 1). This increased connectivity was reflected in higher average degree (AD, 126.93 vs. 91.09), graph density (GD, 0.14 vs. 0.12), and clustering coefficient (CC, 0.58 vs. 0.57) in the night network, indicating denser connections and tighter clustering among microbial taxa.

**Table 1:** Topological properties of the co-occurrence network during diurnal and nocturnal periods.

**Period**	**Diurnal period**	**Nocturnal period**
Total nodes	735	900
Total links	33 474	57 119
Average degree	91.09	126.93
Average path length	1.56	1.50
Graph diameter	5.73	6.74
Graph density	0.12	0.14
Clustering coefficient	0.57	0.58
Betweenness centralization	0.017	0.014
Degree centralization	0.305	0.354
Modularity	0.203	0.143

Analysis of keystone taxa, based on within- (Zi) and among-module (Pi) connectivity, revealed distinct ecological roles and taxonomic compositions for day and night (Fig. 2C). The night network contained 27 key nodes, including 25 connectors and two module hubs, predominantly affiliated with *Flavobacteriales* (13), Blfdi19 (8), *Opitutales* (2), *Rhodobacterales* (2), and *Synechococcales* (2) (Fig. 2D). The day network, in contrast, comprised 37 key nodes, including 36 connectors and 1 module hub, largely belonging to *Synechococcales* (14), *Flavobacteriales* (8), *Bdellovibrionales* (6), SAR86 clade (4), OM190 (2), *Rhodospirillales* (1), *Chitinophagales* (1), and *Burkholderiales* (1) (Fig. 2D). Connectors, acting as bridges between modules, likely facilitate information flow and functional coordination. Module hubs are critical species within their modules, vital for maintaining intra-module ecological processes.

The observed differences suggest that diel light cycles significantly influence microbial community structure and connectivity. Night conditions are associated with more densely connected network structures, implying potentially cooperative relationships facilitating processes like nutrient cycling in the dark. Conversely, day communities exhibit stronger modularity and greater potential for functional specialization within specific clusters, likely related to light-dependent processes like photosynthesis and consumption of phytoplankton-derived organic matter.

### Microbial carbon, nitrogen, and sulfur metabolic functions under the diel light cycle

#### Carbon degradation and fixation

**Figure 3:**
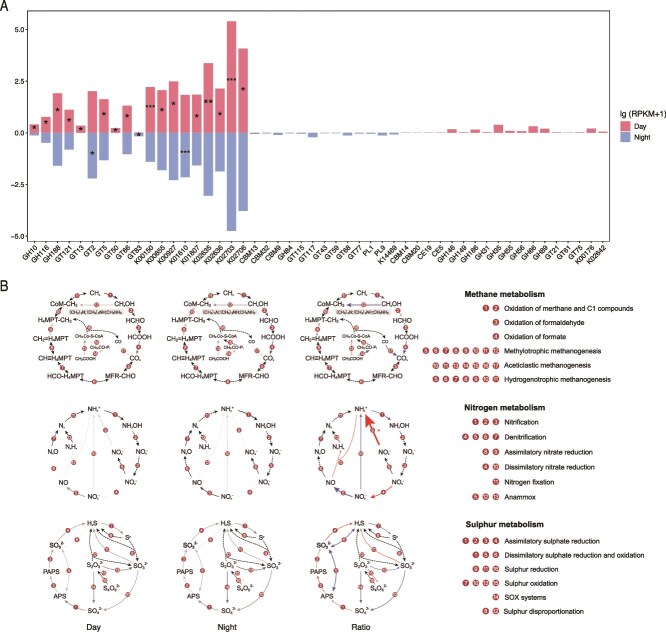
Diel variations in microbial functional gene expression in surface waters of the Pearl River estuary. (A) Differences in transcript abundances of genes involved in carbon degradation and fixation between daytime and nighttime samples. Significance was assessed using the Wilcoxon rank-sum test (*P* < 0.05, ^**^P <0.01, ^***^P < 0.001). Only enzyme families exclusively expressed in either daytime or nighttime samples are shown on the right side; thus, statistical comparisons between diel phases were not performed for these cases. (B) Differential expression analysis of methane-, nitrogen-, and sulfur-related functional genes based on DESeq2. Genes exhibiting an absolute log₂(fold change) ≥ 2 and *P* ≤ 0.05 are marked as significantly different (^*^). Red arrows represent lg [RPKM(day)/RPKM(night)] > 0 and blue arrows represent lg [RPKM(day)/RPKM(night)] < 0. The thickness of each line reflects the magnitude of expression difference, proportional to the absolute value of ratio.

The metatranscriptomic analysis of surface waters in the Pearl River Estuary revealed pronounced diel variations in microbial carbon metabolism and carbohydrate processing. During daytime, carbohydrate-active enzyme (CAZyme) families such as glycosyltransferases (GTs, 29.4%) and glycoside hydrolases (GHs, 28.6%) dominated the functional profile, reflecting active carbohydrate synthesis and degradation. Notably, 13 GH families showed significant differential expression between day and night (*P* < 0.05), with most being enriched during the day, indicating higher carbohydrate turnover when light is available. However, specific enzymes exhibited diel specificity. GH84 was uniquely expressed at night and is associated with hydrolyzing O-GlcNAc modifications on nuclear and cytoplasmic proteins, suggesting a role in nighttime post-translational modification (Fig. 3A). Conversely, carbohydrate esterase families CE5 and CE19 were exclusively active during the day, whereas polysaccharide lyases PL1 and PL9 appeared only at night, implying distinct polysaccharide degradation pathways across the diel cycle.

In terms of autotrophic carbon fixation, genes involved in photosynthesis and the Calvin cycle displayed higher expression during the day, consistent with light-dependent processes. An exception was *pckA* (K01610), which showed higher nighttime transcript abundance, indicating potential roles outside canonical photosynthesis. Additionally, the gene K14469, part of the 3-hydroxypropionate bicycle—a less common autotrophic pathway, was exclusively expressed at night at low levels (average RPKM = 0.19), hinting at specialized autotrophic carbon fixation activity.

#### Methane metabolism

Diel patterns in gene expression revealed dynamic methane metabolism in the studied environment (Fig. 3B). The *mttB* gene (K14083), encoding trimethylamine methyltransferase, showed higher expression at night. This enzyme facilitates the conversion of trimethylamine (TMA) to methyl-coenzyme M, a crucial step potentially promoting methane production via methylotrophic methanogenesis during the dark period. In contrast, the *acss1_*2 gene (K01895), encoding acetyl-CoA synthetase, which is linked to with carbon assimilation pathways, was more highly expressed during the day. This suggests that carbon assimilation processes are favored under light conditions. Further supporting changes in carbon metabolism, the *cooF* (K00196) and *cooS* (K00198) genes, encoding subunits of the carbon monoxide (CO) dehydrogenase complex, were enriched at night. This enrichment suggests an increased rate CO oxidation during the nocturnal hours (Table S3).

#### Nitrogen cycle

Within the nitrogen cycle, several key genes exhibited diel expression patterns indicative of dynamic nitrogen metabolism under light and dark conditions. The *nirA* gene (K00366), which encodes an enzyme involved in assimilatory nitrate reduction—a process converting nitrate to NH₄^+^ for biosynthesis—was significantly more expressed during the day than at night (*P* < 0.05). This suggests that nitrogen assimilation via nitrate reduction is enhanced under light conditions, likely supporting active microbial growth and biosynthesis when energy sources (light) are abundant. Complementary to this, other nitrate reductase genes, including *narI* (K00374), encoding a membrane-bound nitrate reductase subunit, and *napB* (K02568), encoding a periplasmic nitrate reductase subunit, were also relatively enriched during daytime. These genes further support increased nitrate reduction activity in the light period, contributing to nitrogen assimilation pathways. In addition, the gene K20934, encoding a subunit of hydrazine synthase involved in anaerobic NH₄^+^ oxidation (anammox), showed elevated expression during the day (Table S3). This indicates a potential diel regulation of anammox processes, which are typically associated with anaerobic conditions, but here may be modulated by environmental or microbial community shifts in response to the diel cycle. Furthermore, key genes involved in ammonia oxidation, such as *amoA*, *amoB*, and *amoC*, as well as genes related to NO₂^−^ reduction, *nirK* and *nirD*, displayed differential expression between day and night (Fig. 3B). This pattern reflects a diel regulatory effect on nitrification, where ammonia-oxidizing microbes may be more active or regulated by the light–dark cycle, influencing nitrogen fluxes and availability.

#### Sulfur cycle

In the surface waters of the Pearl River Estuary, distinct diel expression patterns were observed for sulfur metabolic functions. Daytime was characterized by elevated transcription of genes involved in assimilatory sulfate reduction (*sat*, *cysN*, *cysD*, *cysC*, *cysH*, *sir*, and *cysJI*), reflecting enhanced anabolic activity and the biosynthesis of sulfur-containing amino acids and cofactors under light conditions. These genes encode enzymes catalyzing the stepwise reduction of sulfate to sulfide, essential for cellular building blocks. Conversely, nighttime saw increased expression of genes related to dissimilatory sulfur reduction (*aprAB* and *dsrAB*) and sulfur oxidation (*fccAB*, *SUOX*, and *soeABC*), suggesting a shift towards anaerobic energy metabolism during the dark period. Specifically, the elevated expression of genes involved in sulfide and sulfite oxidation at night. For thiosulfate oxidation, *TST* and *glpE* genes were more abundant at night, while *tsdA* showed higher expression during the day. Furthermore, SOX system genes (*soxA* to *soxZ*) exhibited overall higher expression at night. Taken together, the data indicate a diel shift in sulfur metabolism. Assimilatory processes for biosynthesis dominate during the day, while energy-generating sulfur oxidation and reduction pathways are more active at night. This temporal partitioning highlights the dynamic nature of microbial sulfur cycling in the estuarine environment.

### Key taxa in carbon, nitrogen, and sulfur metabolism under diel light cycles

**Figure 4:**
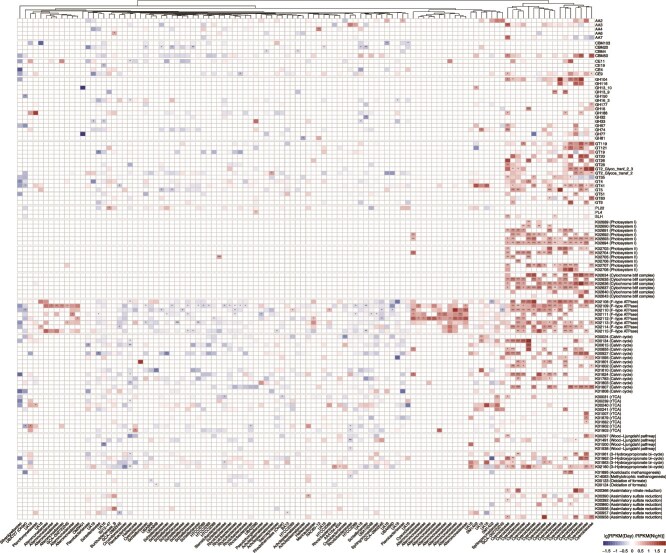
Diel fluctuations in the transcriptional activity of carbon, nitrogen, and sulfur metabolism genes within representative metagenome-assembled genomes (MAGs) were assessed in the Pearl River estuary. The heatmap depicts lg-transformed fold changes [lg (RPKM(day)/RPKM(night))] in gene expression. Asterisks denote statistically significant differences between diurnal and nocturnal groups, as determined by Wilcoxon rank-sum tests (*P* < 0.05, ^**^P < 0.01, ^***^P < 0.001).

To identify the key microbial taxa involved in diel metabolic changes, we reconstructed a total of 786 high- and medium-quality metagenome-assembled genomes (MAGs) from 24 surface water metagenomic samples (Table S4). These MAGs spanned 10 phyla, with the majority belonging to *Pseudomonadota* (39 MAGs), *Bacteroidota* (36 MAGs), and *Cyanobacteriota* (19 MAGs). Notably, all *Cyanobacteriota* MAGs were classified within the family *Cyanobiaceae*, which exhibited significantly elevated transcription of genes associated with photosynthesis, carbon fixation, and organic carbon degradation during the day (Fig. 4).

The high day expression of genes involved in the Calvin cycle, rTCA cycle, Wood–Ljungdahl pathway, and 3-hydroxypropionate bicycle suggests that *Cyanobacteriota* are primary phototrophic autotrophs in the estuarine region, actively driving light-driven carbon assimilation. Additionally, two MAGs exhibited increased expression of aceticlastic methanogenesis genes during daytime, while methylotrophic methanogenesis was more active at night within *Rhodobacteraceae*. The gene expression related to formate oxidation was predominantly contributed by the MAG UBA868, indicating diel shifts in specific organic substrate transformations.

In nitrogen cycling, four MAGs showed higher expression of assimilatory nitrate reduction genes (*nirA*, K00366) during daytime, three of these were *Cyanobacteriota*, indicating their significant role in nitrogen assimilation under light conditions.

Sulfur metabolism exhibited diel variation primarily through changes in assimilatory sulfate reduction gene expression. MAGs with elevated daytime activity included *Cyanobacteriota*, *Halieaceae*, and *Burkholderiaceae_*B, whereas night-active taxa such as BA12 and GCA-2862545 contributed more to sulfur pathways at night. These patterns suggest niche partitioning in sulfur transformations governed by diel light cycles.

Organic carbon degradation enzymes, such as auxiliary activities (AAs), carbohydrate-binding modules (CBMs), glycoside hydrolases (GHs), and glycosyltransferases (GTs), showed pronounced taxon-specific and temporal expression patterns. *Cyanobiaceae*, *Flavobacteriaceae*, and NAC60–12 expressed AAs more strongly during the day, while *Schleiferiaceae* and *Acholeplasmataceae* exhibited higher activity at night.

Regarding photosynthetic complexes, genes encoding Photosystem II, Photosystem I, and cytochrome *b*_6_*f* were predominantly transcribed by *Cyanobiaceae* during daylight hours, with additional contributions from *Steroidobacterales*. Expression of the F-type ATPase differed temporally and taxonomically: daytime expression was primarily observed in *Cyanobiaceae*, *Alteromonadaceae*, *Maricaulaceae*, and HTCC2089 (*Pseudomonadota*), whereas nighttime activity involved diverse taxa including *Flavobacteriaceae*, *Rhodobacteraceae*, *Myxococcota*, *Planctomycetota*, and *Gemmatimonadota*.

Collectively, these findings highlight the centrality of *Cyanobiaceae* in mediating carbon, nitrogen, and sulfur transformations under diel light conditions—demonstrating a clear phototrophic metabolic advantage during the day. The observed temporal partitioning of functional gene expression among different taxa underscores a potential mechanism of metabolic niche segregation driven by diel cycles. Overall, this emphasizes the importance of incorporating diel rhythms into studies of microbially mediated biogeochemical processes to more accurately elucidate their ecosystem-level contributions.

## Discussion

The diel light cycle is a fundamental temporal driver that interacts with concurrent physicochemical processes to shape microbial community dynamics in aquatic ecosystems. While factors such as tides, salinity, and nutrient availability may also influence microbial variability, our sampling design minimized their short-term effects by targeting a hydrodynamically stable period. Consistent with this, environmental parameters showed no significant diel differences (see Section Environmental stability during diel sampling), suggesting that the observed microbial shifts predominantly reflect biological responses to periodic light–dark forcing within this estuarine setting. Nevertheless, in estuarine environments, the mechanisms underlying microbial responses to rhythmic light variation—particularly how light-driven metabolic activity, community interactions, and functional partitioning co-vary over diel phases—remain insufficiently resolved. In this study, we employ high-frequency sampling and integrated multi-omics approaches to elucidate diel shifts in microbial diversity, interaction networks, and metabolic functions within the Pearl River Estuary (PRE). Our results reveal a distinct temporal partitioning of microbial processes: nighttime communities exhibit enhanced α-diversity, more densely connected interaction networks, and a prevalence of heterotrophic metabolic pathways, whereas daytime communities are characterized by *Cyanobacteria*-dominated phototrophic activity. These findings provide crucial insights into the adaptive strategies employed by estuarine microbiomes in response to environmental rhythmicity, addressing a significant knowledge gap in our understanding of microbial ecological responses to diel forcing.

### Diel dynamics of microbial diversity and network structure

Our observation that nighttime α-diversity—measured by richness, Shannon entropy, and inverse Simpson index—exceeds daytime values (Fig. 1B) contrasts with patterns documented in some open ocean systems, where diel fluctuations in diversity are relatively muted [11]. This discrepancy likely reflects the contrasting environmental settings between estuarine and open-ocean systems, where stronger hydrodynamic mixing and nutrient enrichment in the estuary may modulate how light-driven diel rhythms manifest in microbial communities. The elevated nighttime diversity may result from reduced competition for light, which diminishes the resource competition exerted by dominant phototrophic taxa such as *Cyanobacteria*, enabling opportunistic heterotrophs such as *Flavobacteriales* and *Rhodobacterales* to proliferate [48]. Notably, the absence of significant differences in β-diversity between day and night (Fig. 1C) suggests that, despite minor compositional variations over diel cycles, the overall community structure remains relatively stable—a pattern consistent with findings in seasonally stratified lakes [49]. This temporal stability at the taxonomic level likely reflects environmental buffering and the dominance of generalist taxa capable of maintaining population persistence under short-term diel fluctuations. Nevertheless, metatranscriptomic analyses revealed pronounced diel oscillations in functional gene expression, suggesting that microbial metabolic activities can vary dynamically even when community composition appears stable.

Co-occurrence network analyses further illuminate the mechanistic underpinnings of these temporal shifts. Nighttime networks display higher connectivity (average degree: 126.93 vs. 91.09) and clustering coefficients (0.58 vs. 0.57) (Table 1), indicative of stronger metabolic interdependencies under low-light conditions. Key taxa such as *Flavobacteriales* and Blfdi19 emerge as central connectors during nighttime, potentially mediating cross-feeding interactions between heterotrophs and photoheterotrophs [50]. Conversely, daytime networks exhibit greater modularity (modularity score: 0.203 vs. 0.143) (Fig. 2C), reflecting functional compartmentalization driven by cyanobacterial dominance. This modular architecture aligns with models suggesting that light-driven primary producers form tightly integrated clusters to optimize energy capture [6].

Taxonomic analysis of keystone taxa further supports diel functional partitioning. During daylight hours, *Synechococcales* (*Cyanobacteriota*) serve as module hubs, consistent with their roles in carbon fixation and phototrophy (Fig. 2D). At night, however, taxa such as *Flavobacteriales* and *Opitutales* assume keystone positions as network connectors, emphasizing their importance in organic matter degradation and nutrient remineralization [51]. These findings parallel marine studies illustrating diel succession between autotrophs and heterotrophs, driving temporal niche differentiation [12].

### Metabolic partitioning of carbon, nitrogen, and sulfur cycles

Our metatranscriptomic data indicate that microbial carbohydrate metabolism in the estuary follows a diel regulatory pattern, with distinct enzyme assemblages dominating under light and dark conditions. Rather than a simple alternation between anabolic and catabolic states, the data reveal a nuanced division of labor—daytime expression favoring carbohydrate biosynthesis and processing linked to phototrophic activity, while nighttime expression involves alternative polysaccharide degradation (PL1 and PL9). This diel functional differentiation highlights flexible substrate utilization strategies that may optimize carbon turnover and energy conservation under fluctuating light regimes (Fig. 3). During daylight hours, *Cyanobacteria* upregulate genes related to the Calvin cycle (e.g., *rbcL*) and CO_2_ fixation (e.g., *coxL*), reaffirming their role as primary producers (Fig. 4). This pattern aligns with prior research demonstrating diel oscillations in cyanobacterial photosynthesis within estuarine environments [20]. Additionally, genes involved in nitrogen assimilation (e.g., *nirA*) are enriched during the day, indicating a tight coupling between carbon fixation and nitrogen uptake under autotrophic, light-driven conditions [52].

Specifically, analysis of carbohydrate-active enzymes (CAZymes) revealed enzyme-level diel specialization consistent with this broader metabolic framework. During daytime, carbohydrate esterase families such as CE5 and CE19 were predominantly expressed, consistent with active processing of polysaccharides associated with phototrophic production. In contrast, GH84 showed exclusive nighttime expression, suggesting a role in protein-linked carbohydrate modification and organic matter transformation under dark conditions (Fig. 3A). These complementary patterns indicate that carbon processing in the estuarine microbiome follows a temporally partitioned strategy, with phototrophic production during the day and selective heterotrophic transformation at night, rather than a uniform metabolic shift (Fig. 3A). This temporal complementarity supports a refined view of the “microbial carbon pump” hypothesis, wherein diel regulation of specific enzyme families facilitates continuous carbon turnover across light–dark transitions, maintaining metabolic connectivity within the microbial loop [53]. Furthermore, sulfur metabolism displays diurnal shifts: genes for assimilatory sulfate reduction (e.g., *sat*) peak during daylight, while dissimilatory sulfate reduction genes (e.g., *aprAB*) are more active at night, reflecting a trade-off between energy-demanding sulfate reduction in dark, potentially anoxic microenvironments and light-facilitated assimilation of reduced sulfur compounds [16] (Fig. 3B).

Diel expression patterns further underscore metabolic niche differentiation among key taxa. Cyanobacterial MAGs, such as *Synechococcales*, show significant upregulation of photosystem II (PSII) and ATPase genes during the day, facilitating phototrophy. Conversely, heterotrophic MAGs, including *Flavobacteriales*, upregulate genes associated with proteolysis and fermentation at night (Fig. 4). This temporal resource partitioning likely minimizes interspecific competition and contributes to ecosystem stability by allocating metabolic functions across diel niches [1]. At the transcriptomic level, opposite diel expression trends were also evident in key genes mediating nitrogen and sulfur cycling. Assimilatory enzymes such as NirA and CysE were upregulated during the day, consistent with photoautotrophic biomass production, whereas dissimilatory components including AprAB and DsrA showed enhanced nocturnal expression, reflecting energy-yielding heterotrophic metabolism. This inversion reveals a clear functional partitioning between biosynthetic (daytime) and energy-generating (nighttime) processes, suggesting a previously unrecognized diel coupling mechanism among elemental cycles in estuarine microbiomes. Such metabolic alternation likely enhances resource utilization efficiency and contributes to biogeochemical resilience under strong diel light forcing.

Integration of multi-omics datasets further corroborated the ecological roles inferred from community network analysis. Keystone taxa identified from the 16S rRNA gene-based co-occurrence networks showed strong concordance with metabolically active lineages revealed by MAG- and metatranscriptomic analyses. For example, *Synechococcales*, which acted as daytime module hubs in the co-occurrence network, exhibited elevated expression of photosynthesis- and Calvin cycle-related genes, confirming their role as primary phototrophic producers. Conversely, *Flavobacteriales* and *Rhodobacterales*, identified as nighttime network connectors, displayed increased transcription of genes associated with heterotrophic carbon degradation and sulfur oxidation. This cross-validation among datasets demonstrates that topological importance within the microbial network corresponds to actual metabolic activity, reinforcing the robustness of the inferred diel functional partitioning.

### Implications for estuarine biogeochemistry and ecological resilience

The diel partitioning of microbial functions has significant implications for the biogeochemistry of estuarine systems. Daytime autotrophic activity by *Cyanobacteria* enhances net ecosystem productivity through carbon fixation, whereas nocturnal heterotrophic processes facilitate organic matter degradation, nutrient recycling, and potentially influence greenhouse gas fluxes such as CO_2_ and CH_4_. Notably, the nighttime upregulation of methane-cycling genes (e.g., *mttB*) indicates potential links between sulfate reduction and methanogenesis occurring within anoxic microzones (Fig. 3B). These processes are crucial for understanding the global methane budget, as estuaries are recognized both as sources and sinks of atmospheric methane [2].

Furthermore, the temporal segregation of metabolic pathways contributes to ecosystem resilience. By partitioning anabolic and catabolic functions across diel cycles, microbial communities can buffer against environmental stochasticity in resource availability. For example, the daytime accumulation of reduced sulfur compounds, driven by genes such as *cysC* and *cysN*, provides a substrate pool for nighttime sulfide oxidation, helping to maintain redox stability within the water column [15].

Additionally, the observed functional redundancy—where multiple taxa, such as *Synechococcales* and *Flavobacteriales*, contribute to key processes like sulfate reduction—may confer robustness against disturbances. Network analyses reveal that essential metabolic modules are supported by diverse taxa, reducing the risk of systemic collapse under environmental perturbations [54]. This redundancy underscores the importance of community diversity in sustaining ecosystem functions in dynamic estuarine environments.

### Contrasts with freshwater and marine systems

Comparisons with existing literature from freshwater and marine ecosystems reveal both similarities and differences in diel microbial dynamics. The temporal succession between *Cyanobacteria* and heterotrophs, observed in these studies, represents a conserved feature shared with other marine planktonic communities [11]. For instance, a high-frequency metagenomic and metabolic monitoring study in Daya Bay—a neighboring subtropical coastal system—revealed that nearly half of the planktonic microbial taxa, encompassing both phototrophic and heterotrophic lineages, exhibited diel oscillations in community composition and activity [20]. Light intensity and tidal variations were identified as key modulators of these diel patterns, suggesting that temporally phased microbial interactions may be phylogenetically conserved across coastal ecosystems. Likewise, diel rhythmicity in microbial activity is well documented in open-ocean and coastal systems, where bacterioplankton and phytoplankton display synchronized oscillations in gene expression and metabolism tightly coupled to light–dark cycles [7, 55, 56]. Collectively, these studies demonstrate a functional partitioning of metabolic processes across the diel cycle: phototrophs dominate daytime anabolic activities, whereas heterotrophs engage in nocturnal catabolism, promoting efficient resource recycling within the microbial loop. However, the amplitude of diel metabolic observed in estuarine systems tends to be amplified than that in open-ocean or lacustrine environments, reflecting the inherently dynamic physicochemical setting of estuaries, where background hydrodynamic mixing and episodic nutrient renewal may modulate how microbial communities respond to light forcing over time [16]. Rather than attributing this directly to short-term hydrodynamic or nutrient fluctuations—which showed no significant diel variation in our dataset—we suggest that the inherently variable physicochemical background of estuaries provides a broader regulatory context that modulates the stability and amplitude of microbial diel rhythms over time. While our statistical analyses confirmed no significant diel variation in hydrodynamic or physicochemical parameters (e.g., tidal height, salinity, temperature), the Pearl River Estuary remains a highly heterogeneous system over broader spatial and temporal scales. Such background variability—arising from gradients in depth, nutrient availability, and mixing intensity—may modulate the amplitude or stability of diel transcriptional rhythms rather than directly driving short-term differences. Previous work in the PRE has demonstrated pronounced spatial heterogeneity in microbial functional gene composition linked to depth and nutrient gradients [19], providing an ecological context for the diel functional partitioning observed in this study.

### Limitations and future directions

While our study offers valuable new insights into diel microbial dynamics in estuarine systems, several limitations should be acknowledged. Firstly, the 48-hour sampling window provides only a snapshot of diel fluctuations; extending the temporal scope to encompass seasonal and interannual variability would enhance understanding of long-term patterns and drivers. Secondly, the functional analyses were primarily based on bulk community measurements, which limit resolution regarding individual taxa. Employing single-cell techniques, such as single-cell genomics or transcriptomics, could reveal intraspecific heterogeneity in gene expression and activity. Thirdly, the potential impacts of biological interactions—including viral-mediated lysis, protistan grazing, fungal parasitism, and macrofaunal disturbance—on microbial turnover and community composition remain unexplored in this context. These processes can strongly influence microbial diversity and metabolic coupling in estuarine ecosystems and warrant further investigation through combined metatranscriptomic and experimental approaches. Integrating viromics approaches and advanced methods like nanoscale secondary ion mass spectrometry (NanoSIMS) with targeted methods for protists, fungi, and macrofauna—such as single-cell genomics, metatranscriptomics, and microcosm experiments—could provide insights into viral-host interactions and their role in shaping diel microbial processes. Although this study focused on a single representative station to ensure high temporal resolution, we acknowledge that spatial variability—such as differences in water depth, salinity regimes, and anthropogenic inputs—may influence microbial diel patterns. Future research combining spatially distributed diel sampling and hydrodynamic modeling will be essential to generalize the observed mechanisms across the entire estuary. Addressing these gaps in future research will deepen our understanding of the complexity and resilience of estuarine microbial ecosystems.

## Conclusion

This study demonstrates that diel light cycles drive pronounced temporal portioning in microbial community structure and function in the Pearl River Estuary. Through integrated multi-omics approaches, we revealed that nighttime periods foster higher α-diversity and enhanced network connectivity, facilitating heterotrophic processes such as organic matter degradation and dissimilatory sulfur reduction. In contrast, daytime communities are dominated by *Cyanobacteria*, which upregulate photoautotrophic pathways including carbon fixation and nitrogen assimilation. These findings establish a direct mechanistic link between light-driven diel oscillations and microbial adaptive strategies, highlighting how temporal dynamics underpin biogeochemical resilience in estuarine ecosystems. Our work provides a high-resolution framework for understanding microbial diel rhythms and offers a foundation for future studies on ecosystem-scale modeling and climate changes impacts on coastal biogeochemical cycles.

## Supplementary Material

Table_S1_ycaf216

Table_S2_ycaf216

Table_S3_ycaf216

Table_S4_ycaf216

## Data Availability

All sequencing data, including amplicons, metagenomes, and metatranscriptomes, are accessible at the NCBI-SRA under the project number PRJNA1067541.
